# Crystal structure of 4,5-dimethyl-1,3-dioxol-2-one

**DOI:** 10.1107/S2056989022009239

**Published:** 2022-10-11

**Authors:** Chandru P. Chandrasekaran, James P. Donahue

**Affiliations:** a Department of Chemistry and Biochemistry, Lamar University, Beaumont, TX 77710, USA; b Department of Chemistry, Tulane University, 6400 Freret Street, New Orleans, Louisiana 70118-5698, USA; Dublin City University, Ireland

**Keywords:** crystal structure, carbonate, dioxolene ligand

## Abstract

The planar title compound 4,5-dimethyl-1,3-dioxol-2-one, C_5_H_6_O_3_, **1**, crystallizes with its mol­ecular *C*_2_ axis coincident with a crystallographic mirror plane in space group *P*2_1_/*m*. In the plane defined by the *b* axis and an *ac* face diagonal, anti­parallel linear strands of **1**, formed by simple translation, associate to form sheets with close H⋯H and O⋯O inter­molecular contacts. Between the sheets, parallel strands of **1** place the carbonyl O atom near the five-membered ring centroid of a neighboring mol­ecule with close O⋯O and O⋯C contacts.

## Abstract 2

4,5-Dimethyl-1,3-dioxol-2-one crystallizes on a mirror plane in *P*2_1_/*m* and forms layered sheets that comprise anti­parallel linear strands. Close O⋯H, C⋯O and C⋯C inter­molecular contacts are formed between strands and sheets.

## 1. Chemical context

**Figure 1:**
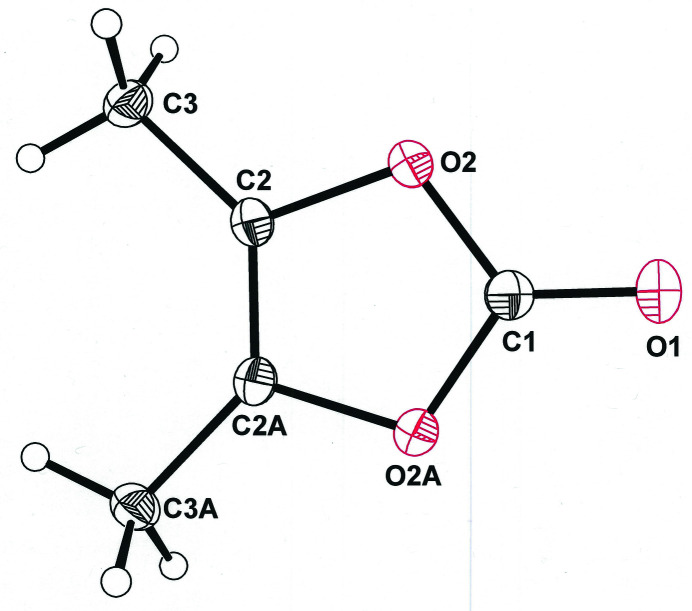
Displacement ellipsoid plot (50% probability) of 4,5-dimethyl-1,3-dioxol-2-one (**1** [Chem scheme1]) with non-H atom labeling.

4,5-Dimethyl-1,3-dioxol-2-one, **1** (Fig. 1 ▸ and scheme[Chem scheme1]), is a simple derivative of vinyl­ene carbonate, **2a** [Chem scheme1], that has attracted recent attention as a key component of non-aqueous electrolyte blends for advanced Li ion batteries (Park *et al.*, 2021 ▸; Liu *et al.*, 2017 ▸; Kotani & Kadota, 2016 ▸; Xu *et al.*, 2010 ▸). Its 4-chloro­methyl and 4-bromo­methyl derivatives, **3** [Chem scheme1], have significance in the pharmaceutical industry as building elements in the preparation of antibiotics such as prulifloxacin (Cao *et al.*, 2013 ▸), cefuroxime variants (Webber, 1987 ▸), and ampicillin (Sakamoto *et al.*, 1984 ▸; Xiao, 2004 ▸). In principle, by analogy to the usefulness that the related 4,5-dimethyl-1,3-di­thiol-2-one, **4a**, enjoys as a masked form of the di­methyl­dithiol­ene ligand (Chandrasekaran *et al.*, 2009 ▸), **1** [Chem scheme1] could function as a protected form of the dimethyldioxolene(2−) ligand, **5** [Chem scheme1], that is liberated by straightforward base hydrolysis. 
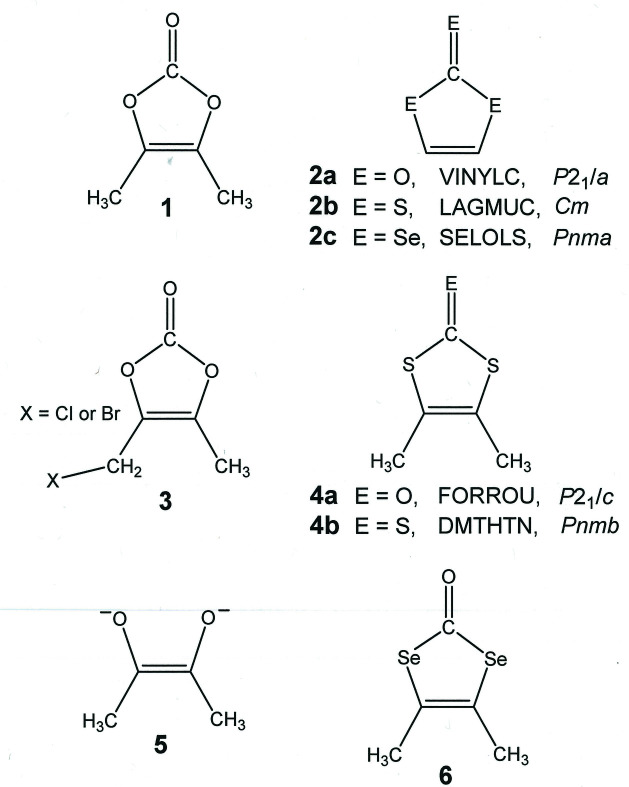


Although a few coordination complexes with the di­methyl­dioxolene ligand are known, they have been prepared by oxidative addition of the corresponding *α*-diketone to a low-valent metal precursor (Chisholm *et al.*, 1983 ▸) or by an obscure route involving the reductive coupling of CO(*g*) with methyl ligands (Hofmann *et al.*, 1985 ▸). This context of demonstrated usefulness and unrealized, but plausible, possibility for **1** [Chem scheme1] persuaded us to undertake a study of its utility as a dioxolene ligand precursor. In an early research stage, serendipitously obtained diffraction-quality crystals of **1** [Chem scheme1] provided an opportunity for characterization by X-ray diffraction, details of which are reported herein.

## 2. Structural commentary

Compound **1** crystallizes in the monoclinic space group *P*2_1_/*m* upon a crystallographic mirror plane that coincides with the carbonyl bond (Fig. 1 ▸).

## 3. Supra­molecular features

**Figure 2:**
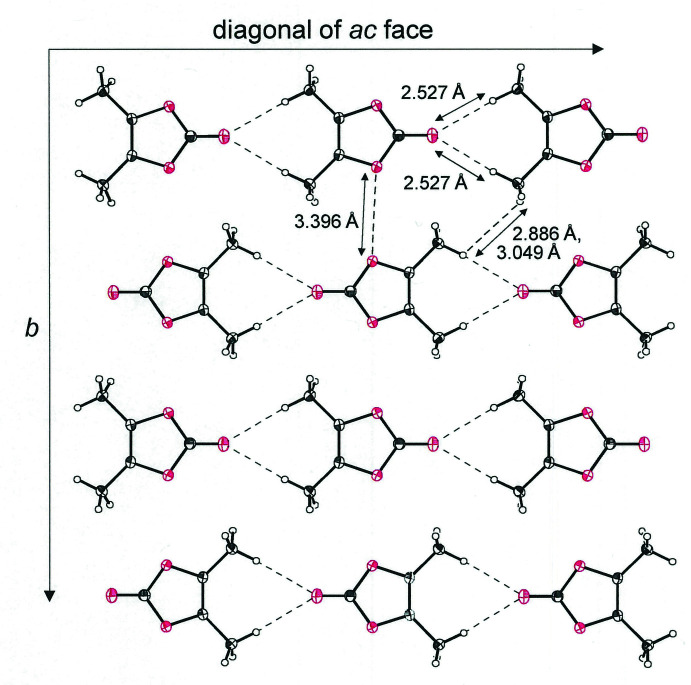
Packing diagram for 4,5-dimethyl-1,3-dioxol-2-one (**1** [Chem scheme1]) illustrating the sheetlike arrangement in the plane defined by the *b* axis and an *ac* face diagonal. Displacement ellipsoids are at the 50% level, and closest inter­molecular contacts are indicated.

**Figure 3:**
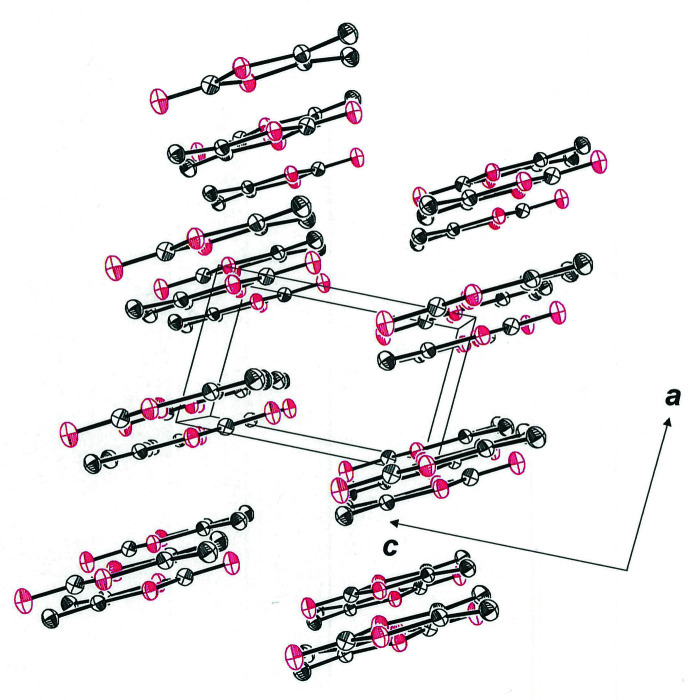
Cell packing diagram of **1** [Chem scheme1] with a view along the *b* axis of the cell. Displacement ellipsoids are drawn at the 50% level, and all H atoms are omitted for clarity.

Mol­ecules of **1** [Chem scheme1] are aligned as one-dimensional strands by simple translation along one of the diagonals of the *ac* face of the unit cell (Fig. 2 ▸). The C=O oxygen atom of **1** [Chem scheme1] forms close contacts of 2.53 Å with the hydrogen atoms from each of the methyl groups of the mol­ecule aligned before or behind (Table 1 ▸), at a distance that approaches the sum of the van der Waals radii of the elements (Batsanov, 2001 ▸). These strands are further organized into two-dimensional sheets through side-by-side placement but with an alternating orientation of the polarized, carbonyl end of the mol­ecules (Fig. 2 ▸). The *b* axis defines the 2nd dimension of these sheets. Between strands within these sheets, inter­atomic H⋯H separations are 2.89 and 3.05 Å, while nearest O⋯O distances between rings are 3.3962 (13) Å. Fig. 3 ▸ presents a perspective of these sheets that is approximately along the *b* axis of the cell such that the close stacking between them is visible.

**Figure 4:**
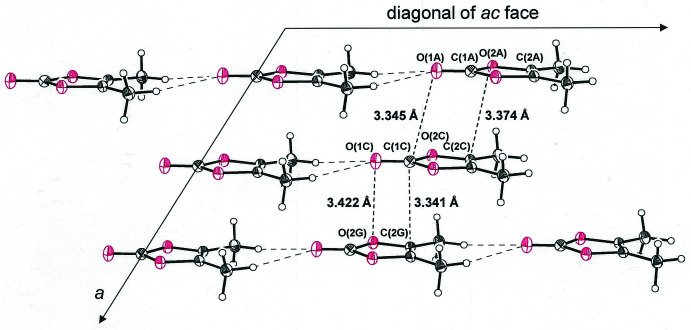
View of the cell packing arrangement in **1** [Chem scheme1] depicting the closest inter­molecular contacts between linear strands extending in the direction of a diagonal to the *ac* face. Displacement ellipsoids are at the 50% level.

**Figure 5:**
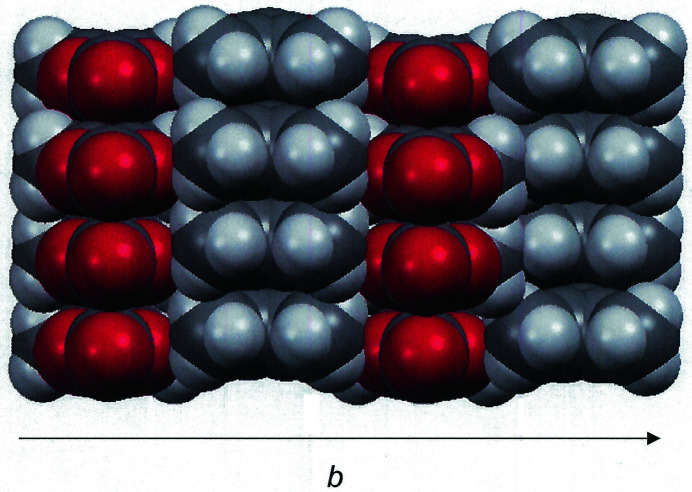
Space-filling plot of the unit-cell packing in **1** [Chem scheme1] as viewed along the length of the linear strands, which are orthogonal to the paper plane.

An alternative description of the third dimension of the packing is that the one-dimensional strands noted above translate as a whole along the *a* axis of the cell with an offset that places the carbonyl oxygen atom of one mol­ecule of one strand near the five-membered ring centroid of a neighboring mol­ecule (Fig. 4 ▸). In contrast to the *intra*sheet strands depicted in Fig. 2 ▸, which are anti­parallel, the neighboring *inter*sheet strands are all oriented in the same direction. The closest inter­molecular C⋯C and C⋯O contacts between these parallel strands are the C_c_⋯C_o_ separation of 3.3413 (16) Å, the O_c_⋯C_c_ spacing of 3.3452 (18) Å, and the O_r_⋯C_o_ distance of 3.3742 (13) Å (c = carbonyl, o = olefin, r = ring). It is likely that the electrostatic inter­actions of polarized bonds, *e.g.*, placement of the negative end of the ^δ(–)^O=C^δ(+)^ carbonyl dipole above the positive end of the same bond in the sheet below, exert a decisive role in guiding the organization and spacing of one mol­ecular plane over another. An end-on view of these sheets in space-filling presentation mode emphasizes the packing efficiency imposed by these cumulative inter­molecular inter­actions (Fig. 5 ▸).

## 4. Database survey

**Figure 6:**
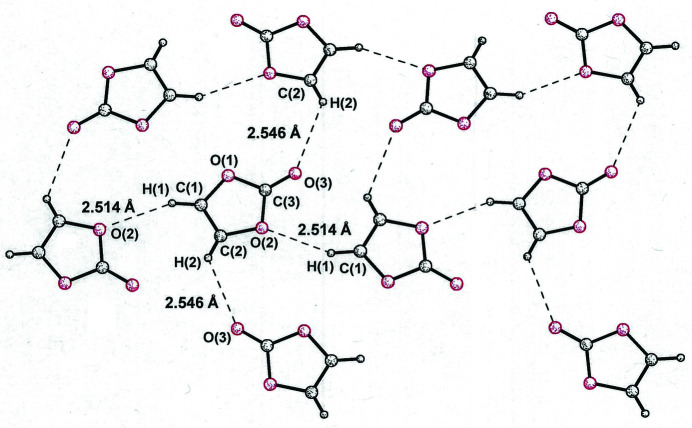
Ball and stick representation of the sheetlike arrangement in **2a** [Chem scheme1], where mol­ecules (left-to-right) are related by a glide-plane operation rather than simple translation. Closest inter­molecular contacts are illustrated.

Of the relatively few vinyl­ene carbonates that have been structurally characterized, only **1** [Chem scheme1] and the parent compound **2a** [Chem scheme1] (Cser, 1974 ▸) are simple, symmetrically substituted variants. All other structurally identified compounds bearing this moiety are more complex organic mol­ecules that have been prepared and studied as angiotensin II receptor blockers (Yanagisawa *et al.*, 1996 ▸; Dams *et al.*, 2015 ▸; Zhang *et al.*, 2017 ▸). Despite its ostensible similarity to **1** [Chem scheme1], compound **2a** [Chem scheme1] crystallizes in a rather different fashion. Although arranged into extended sheets, which also contain the *b* axis, mol­ecules of **2a** [Chem scheme1] are not organized into discernible linear strands but instead are twisted relative to their neighbors so as to accommodate multiple C—H⋯O hydrogen-bonding inter­actions (Fig. 6 ▸). A glide plane, rather than simple translation, relates one mol­ecule of **2a** [Chem scheme1] to another in the horizontal direction (Fig. 6 ▸). As their different space groups would necessitate, the packing arrangements for vinyl­ene tri­thio­carbonate (**2b** [Chem scheme1], CSD refcode LAGMUC; Mereiter & Rosenau, 2005 ▸) and vinyl­ene tris­elenolate (**2c** [Chem scheme1], SELOLS; Lyubovskaya *et al.*, 1976 ▸) contrast greatly with **2a** [Chem scheme1]. The former reveals linear strands of mol­ecules arranged in sheets with a parallel orientation of all strands. Inter­molecular π–π stacking inter­actions appear to be the decisive packing force between sheets. The latter, when viewed along the *b* axis of the cell, reveals a herringbone-like pattern in the arrangement of mol­ecules.

Compounds similar to **1** [Chem scheme1] with methyl substituents at the 4 and 5 positions of the ring include **4a** [Chem scheme1], already noted, and the all-sulfur form, **4b** [Chem scheme1] (DMTHTN; Smith & Luss, 1980 ▸). Compound **4b** [Chem scheme1] occurs in the same space group (No. 62, *Pnma*) as **2c** [Chem scheme1] with a qualitatively similar packing arrangement that differs in having the herringbone pattern visible when viewed along the cell’s *a* axis. Compound **4a** [Chem scheme1] crystallizes in *P*2_1_/*c* on a general position with similar generalities of description pertinent to its packing pattern as found for **2a** [Chem scheme1]. However, adjacent strands of **4b** that are generated by the glide plane operation are slightly out of plane relative to one another. The selenium analogue (**6** [Chem scheme1]) of **1** [Chem scheme1] and **4a** [Chem scheme1] has not been characterized crystallographically but is a target of current study in our laboratory.

## 5. Synthesis and crystallization

The sample of 4,5-dimethyl-1,3-dioxol-2-one used in this study was purchased from AK Scientific, Inc. and recrystallized by evaporation of a MeOH solution from a test tube at room temperature.

## 6. Refinement

Crystal data, data collection and structure refinement details are summarized in Table 2 ▸. Hydrogen atoms are added in calculated positions and refined with isotropic displacement parameters that are approximately 1.5 times those of the carbon atom to which they are attached. The C—H distances are fixed at 0.98 Å.

## Supplementary Material

Crystal structure: contains datablock(s) I, global. DOI: 10.1107/S2056989022009239/gg4011sup1.cif

Supporting information file. DOI: 10.1107/S2056989022009239/gg4011Isup2.cml

CCDC reference: 2207848

Additional supporting information:  crystallographic information; 3D view; checkCIF report

## Figures and Tables

**Table 1:** Hydrogen-bond geometry (Å, °)

*D*—H⋯*A*	*D*—H	H⋯*A*	*D*⋯*A*	*D*—H⋯*A*
C3—H3*A*⋯O1^i^	0.98	2.53	3.4976 (13)	171

Symmetry code: (i) 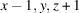 .

**Table 2:** Experimental details

Crystal data	
Chemical formula	C_5_H_6_O_3_
*M*_r_	114.10
Crystal system, space group	Monoclinic, *P*2_1_/*m*
Temperature (K)	100
*a*, *b*, *c* (Å)	3.8283 (10), 10.972 (2), 6.1096 (10)
β (°)	93.523 (2)
*V* (Å^3^)	256.15 (10)
*Z*	2
Radiation type	Mo *K*α
μ (mm^−1^)	0.12
Crystal size (mm)	0.27 × 0.21 × 0.16

Data collection	
Diffractometer	Bruker SMART APEX
Absorption correction	Multi-scan (*SADABS*; Krause *et al.*, 2015 ▸)
*T*_min_, *T*_max_	0.967, 0.980
No. of measured, independent and observed [*I* > 2σ(*I*)] reflections	2310, 636, 617
*R*_int_	0.027
(sin θ/λ)_max_ (Å^−1^)	0.667

Refinement	
*R*[*F* ^2^ > 2σ(*F* ^2^)], *wR*(*F* ^2^), *S*	0.030, 0.077, 1.10
No. of reflections	636
No. of parameters	42
H-atom treatment	H-atom parameters constrained
Δρ_max_, Δρ_min_ (e Å^−3^)	0.30, −0.21

Computer programs: *SMART* (Bruker, 2000 ▸), *SAINT-Plus* (Bruker, 2004 ▸), *SHELXT* (Sheldrick, 2015*a* ▸), *SHELXL* (Sheldrick, 2015*b* ▸), and *SHELXTL* (Sheldrick, 2008 ▸).

## supplementary crystallographic information

### Crystal data

**Table:**

C_5_H_6_O_3_	*F*(000) = 120
*M**_r_* = 114.10	*D*_x_ = 1.479 Mg m^−^^3^
Monoclinic, *P*2_1_/*m*	Mo *K*α radiation, λ = 0.71073 Å
*a* = 3.8283 (10) Å	Cell parameters from 2113 reflections
*b* = 10.972 (2) Å	θ = 3.2–28.3°
*c* = 6.1096 (10) Å	µ = 0.12 mm^−^^1^
β = 93.523 (2)°	*T* = 100 K
*V* = 256.15 (10) Å^3^	Block, white
*Z* = 2	0.27 × 0.21 × 0.16 mm

### Data collection

**Table:**

Bruker SMART APEX diffractometer	636 independent reflections
Radiation source: fine-focus sealed tube	617 reflections with *I* > 2σ(*I*)
Graphite monochromator	*R*_int_ = 0.027
π and ο scans	θ_max_ = 28.3°, θ_min_ = 3.3°
Absorption correction: multi-scan (SADABS; Krause *et al.*, 2015)	*h* = −4→4
*T*_min_ = 0.967, *T*_max_ = 0.980	*k* = −14→14
2310 measured reflections	*l* = −8→8

### Refinement

**Table:**

Refinement on *F*^2^	Secondary atom site location: difference Fourier map
Least-squares matrix: full	Hydrogen site location: inferred from neighbouring sites
*R*[*F*^2^ > 2σ(*F*^2^)] = 0.030	H-atom parameters constrained
*wR*(*F*^2^) = 0.077	*w* = 1/[σ^2^(*F*_o_^2^) + (0.0345*P*)^2^ + 0.0767*P*] where *P* = (*F*_o_^2^ + 2*F*_c_^2^)/3
*S* = 1.10	(Δ/σ)_max_ < 0.001
636 reflections	Δρ_max_ = 0.30 e Å^−^^3^
42 parameters	Δρ_min_ = −0.21 e Å^−^^3^
0 restraints	Extinction correction: SHELXL, Fc^*^=kFc[1+0.001xFc^2^λ^3^/sin(2θ)]^-1/4^
Primary atom site location: structure-invariant direct methods	Extinction coefficient: 0.22 (2)

### Special details

**Table:**

Geometry. All esds (except the esd in the dihedral angle between two l.s. planes) are estimated using the full covariance matrix. The cell esds are taken into account individually in the estimation of esds in distances, angles and torsion angles; correlations between esds in cell parameters are only used when they are defined by crystal symmetry. An approximate (isotropic) treatment of cell esds is used for estimating esds involving l.s. planes.
Refinement. Refinement of F^2^ against ALL reflections. The weighted R-factor wR and goodness of fit S are based on F^2^, conventional R-factors R are based on F, with F set to zero for negative F^2^. The threshold expression of F^2^ > 2sigma(F^2^) is used only for calculating R-factors(gt) etc. and is not relevant to the choice of reflections for refinement. R-factors based on F^2^ are statistically about twice as large as those based on F, and R- factors based on ALL data will be even larger. Hydrogen atoms were added in calculated positions and refined with isotropic displacement parameters that are approximately 1.5 times those of the carbon atom to which they are attached. The C–H distances assumed were 0.98 Å.

### Fractional atomic coordinates and isotropic or equivalent isotropic displacement parameters (Å^2^)

**Table:**

	*x*	*y*	*z*	*U*_iso_*/*U*_eq_	
O1	0.1654 (3)	0.250000	0.67266 (16)	0.0247 (3)	
O2	−0.08340 (16)	0.35049 (6)	0.94742 (10)	0.0174 (2)	
C1	0.0147 (3)	0.250000	0.8382 (2)	0.0177 (3)	
C2	−0.2444 (2)	0.31041 (8)	1.13620 (13)	0.0156 (2)	
C3	−0.3662 (2)	0.40519 (8)	1.28542 (15)	0.0190 (2)	
H3A	−0.479500	0.366409	1.407001	0.028*	
H3B	−0.534168	0.458611	1.204840	0.028*	
H3C	−0.165882	0.453408	1.343402	0.028*	

### Atomic displacement parameters (Å^2^)

**Table:**

	*U*^11^	*U*^22^	*U*^33^	*U*^12^	*U*^13^	*U*^23^
O1	0.0293 (6)	0.0271 (5)	0.0186 (5)	0.000	0.0091 (4)	0.000
O2	0.0204 (4)	0.0160 (4)	0.0162 (4)	−0.0002 (2)	0.0045 (2)	0.0011 (2)
C1	0.0185 (6)	0.0176 (6)	0.0169 (6)	0.000	0.0008 (4)	0.000
C2	0.0145 (4)	0.0180 (5)	0.0145 (4)	−0.0006 (3)	0.0020 (3)	0.0009 (3)
C3	0.0206 (5)	0.0168 (4)	0.0199 (4)	0.0004 (3)	0.0044 (3)	−0.0027 (3)

### Geometric parameters (Å, º)

**Table:**

O1—C1	1.1949 (16)	C2—C3	1.4773 (12)
O2—C1	1.3537 (10)	C3—H3A	0.9800
O2—C2	1.4110 (10)	C3—H3B	0.9800
C2—C2^i^	1.3257 (18)	C3—H3C	0.9800
			
C1—O2—C2	107.30 (7)	C2—C3—H3A	109.5
O1—C1—O2^i^	125.46 (5)	C2—C3—H3B	109.5
O1—C1—O2	125.46 (5)	H3A—C3—H3B	109.5
O2^i^—C1—O2	109.07 (10)	C2—C3—H3C	109.5
C2^i^—C2—O2	108.16 (4)	H3A—C3—H3C	109.5
C2^i^—C2—C3	134.74 (5)	H3B—C3—H3C	109.5
O2—C2—C3	117.09 (7)		
			
C2—O2—C1—O1	−178.43 (12)	C1—O2—C2—C2^i^	−0.72 (8)
C2—O2—C1—O2^i^	1.19 (12)	C1—O2—C2—C3	178.50 (8)

Symmetry code: (i) *x*, −*y*+1/2, *z*.

### Hydrogen-bond geometry (Å, º)

**Table:**

*D*—H···*A*	*D*—H	H···*A*	*D*···*A*	*D*—H···*A*
C3—H3*A*···O1^ii^	0.98	2.53	3.4976 (13)	171

Symmetry code: (ii) *x*−1, *y*, *z*+1.
